# Forensic species identification: practical guide for animal and plant DNA analysis

**DOI:** 10.1007/s00414-024-03284-2

**Published:** 2024-07-10

**Authors:** Beatrice Corradini, Denise Gianfreda, Gianmarco Ferri, Francesca Ferrari, Ilaria Borciani, Anna Laura Santunione, Rossana Cecchi

**Affiliations:** 1 Department of Biomedical, Metabolic and Neural Sciences, Institute of Legal Medicine, University of Modena and Reggio Emilia, Modena, Italy; 2 University - Hospital Polyclinic of Modena, Modena, Italy

**Keywords:** Species identification, Forensic botany, Crime scene investigations, Non-human DNA analysis, DNA barcoding, Forensic evidence

## Abstract

The importance of non-human DNA in the forensic field has increased greatly in recent years, together with the type of applications. The molecular species identification of animal and botanical material may be crucial both for wildlife trafficking and crime scene investigation. However, especially for forensic botany, several challenges slow down the implementation of the discipline in the routine.

Although the importance of molecular analysis of animal origin samples is widely recognized and the same value is acknowledged to the botanical counterpart, the latter does not find the same degree of application.

The availability of molecular methods, especially useful in cases where the material is fragmented, scarce or spoiled preventing the morphological identification, is not well known. This work is intended to reaffirm the relevance of non-human forensic genetics (NHFG), highlighting differences, benefits and pitfalls of the current most common molecular analysis workflow for animal and botanical samples, giving a practical guide. A flowchart describing the analysis paths, divided in three major working areas (inspection and sampling, molecular analysis, data processing and interpretation), is provided. More real casework examples of the utility of non-human evidence in forensic investigations should be shared by the scientific community, especially for plants. Moreover, concrete efforts to encourage initiatives in order to promote quality and standardization in the NHFG field are also needed.

## Introduction

The interest of the forensic community for non-human biological material has grown greatly in the last decades. Consequently, molecular analysis of non-human evidences increasingly gave rise to a new specialized branch of forensic science, known as non-human forensic genetics (NHFG), which deals with the identification of taxonomic species for forensic and legal purpose [1]. The two main responses that a molecular analysis can provide are: species identification (*what is it?*) and individual identification (*who is it?*) [2].

In turn, forensic species identification could concern two fields of application both for plant and animal contexts: the first involves International Wildlife Trafficking (IWT) and the second embraces the examination of evidences related to crime scene investigation (CSI) [3, 4].

Specifically, the illegal wildlife trade could be considered the most prosecuted crime in many geographical areas (tropical or subtropical) due the widespread presence of protected or prohibited species [5–7].

For what concern crime scene investigation, the use of non-human biological traces (NHBT) [8, 9] as investigative tool is still mostly underestimated, particularly for botany [10].

A lot of biological traces could be helpful to the resolution of both indoor and outdoor crimes and several casework examples document the successful use of this type of evidence as investigative lead [11]. Despite the proven importance of NHBT in forensic investigations, these evidences are often discarded or not properly considered probably due to a little knowledge and awareness among practitioners and law enforcement about the use, the potential and the applicability of non-human species identification in forensic cases. The investigators are not quite aware that they can count on the existence of molecular methods which can offer help and investigative strategy, even in cases where the material is fragmented or spoiled to such an extent that morphological identification by observation is not possible. While the importance of the animal counterpart is already more fixed leading to a deeper know-how in the field, for plants it’s a more complex matter. Plants require more attention and training to bring its importance to light, as emerges in detail from the recent work on the state of the art of forensic botany by Oliveira et al. [10]. The authors’ experience, together with the challenges encountered [12–17], has allowed to identify some of the critical points when approaching to the classical molecular procedure.

This work aims to briefly illustrate to forensic geneticists, that often does not possess taxonomic notions, the multiphase process behind the molecular identification of animal and botanical species, and to provide a simplified and explanatory path to refer when dealing with NHBT. Differences between the animal and plant species identification context are highlighted. The forensic diagnosis of species, indeed, involves a series of decision-making phases that not only depend on the type of biological material sampled, but also on the type of investigation requested.

**Fig. 1:**
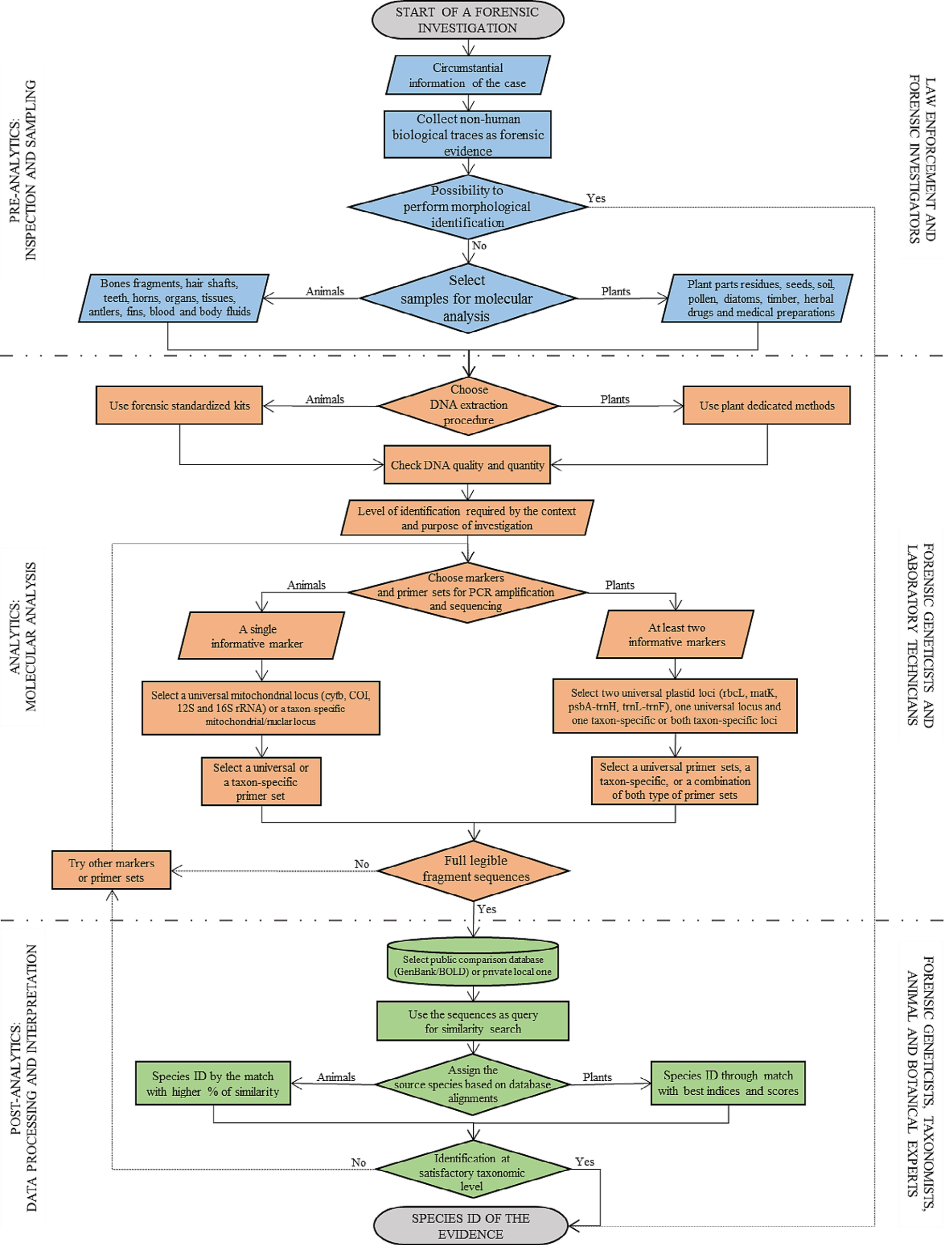
Flowchart highlighting the forensic species identification (species ID) workflow divided into three areas: blue for the pre-analytical phase, orange for analytics, and green for the post-analytics. For each of the three colored areas, the actions carried out at that stage and those who carry out and manage the working activities have been described

For this purpose, a flowchart divided into three major working areas (pre-analytics, analytics, post-analytics) is presented, describing the different possible routes and criticalities to keep in mind (Fig. 1).

## Inspection and sampling *(flowchart blue area)*

When a forensic investigation starts, the first step is the proper recognition of the non-human forensic evidence. This is a critical point as procedural gaps in this phase causes the loss of this type of evidence due to a lack of habit of recognition.

It’s of fundamental importance to document the crime scene via careful collection of photographic and descriptive documentation. Therefore, all forensic experts who operate at the crime scene must be aware and adequately trained on what they must look for and find. Once identified, the biological material to be subjected to morphological identification and molecular analysis should be sampled. This phase must be conducted and guided based on considering the specific investigative context and on the circumstantial information available.

In wildlife forensic science, the type of collectable NHBT is wide-ranging and it can be found in the form of whole organisms, body parts, products and derivatives of both of animal and botanical origins. For animals, body parts could involve bones, antlers, horns, teeth, organs as well as degraded or processed tissues [8, 9, 18–26]. For plants, the common parts encountered in wildlife are often in modified forms such as timber logging, leaves or roots fragments, powders, herbal preparations and medicinal derivatives, seized drugs or protected exotic species which do not allow the recognition of the belonging species at a visual level [27–42].

Wildlife species analysis are especially implemented in tropical areas, where the investigated species inhabit, or in import/export transit geographical areas that serve as commercial hub for illegal trade listed in the Convention on International Trade in Endangered Species of Wild Fauna and Flora (CITES) [6, 7]. These analyses are mainly entrusted to state legal forensic laboratory, international control government services or non-forensic specialized university academics laboratory.

Species identification is often a question of distinguishing between traces of human or non-human origin, in order to locate the human material and eventually proceed with the individualization. This is the case when human and animal bone remains are found mixed [12]. Inspections in animal attacks situations can lead to the collection of animal evidences in correspondence of injuries and wounds of the victim: the species of origin could be identified through DNA-based technique in that type of sample [22–24].

Botanical material can be found in the form of intact parts of a plant such as leaves, stems, roots, flowers, bark, seeds, pollen or more often as fragmented portions [10]. Plant residues can be collected from the body (e.g., skin, under fingernails, inside mouth) and clothing, shoes of victims or offenders, carpets, floor, ground, vehicles (e.g. inner, wheels) or items involved in the crime. Plants can provide forensic evidence in several judicial scenarios where DNA-based species identification led to crucial investigative clues. This may concern the establishment of links between victims, perpetrators and places [27–36], identification of burial sites [37, 38], determine the cause of death [39–41], the pointing out of displacement of a corpse peri-mortem or post-mortem [42], estimation time since death (post mortem interval, PMI) [40], individualization of toxins in stomach content [40], highlight items or weapon involved in violent crime [42].

For example, trace evidence could help in rebuilding the displacements as demonstrated by some studies according to which fragments of bryophytes can easily remain attached to shoes and clothes, and even if the plant has been fragmented, their DNA can be analysed [33, 43]. Due to their ubiquitous presence, they could be unintentionally transported from the crime scene environment to the victims, shoes, nails or skin of the people involved in the crime. Another source of botanical evidence, almost never considered for molecular analysis, is the stomach and intestinal contents. Plant traces, derived from a meal of vegetables or legumes or seeds, cause to their nature, are potentially detectable for more time, compared to traces of animal meal, and can give indications of the timing of death [40].

Improving standardization in the field of forensic botany could lead to a decrease in the events of contamination and loss of material, strengthening the value and usefulness of DNA analysis in investigative contexts and it can help forensic investigator in a little-known context to conduct an analysis which do not belong to the routine.

These practical indications for non-human evidences collection can be found in the Best Practice Manual issued by ENFSI [8]. The detection and sampling of traces of animal biological material as forensic evidence is far more represented in judicial contexts, supposedly because their similarity in appearance and handling with human origin traces, is largely represented in literature [1, 2]. The lack of specific guidelines and the completely different nature of the botanical traces, makes the recognition and the subsequently sampling and managing of plant material harder. Some practical suggestions about this field are provided in literature [10, 29, 44].

Improving non-human DNA analysis standardization, especially for forensic botany, could help to strength the value and usefulness of DNA analysis in investigative contexts and encouraging non-routine supportive forensic analysis.

## Molecular analysis *(flowchart orange area)*

The current gold standard approach for forensic molecular species identification implies DNA extraction and quantitation, PCR amplification and Sanger sequencing of high inter-species variable regions through conserved primers, followed by sequence alignment and comparison with those available in the reference databases. The DNA extraction is the first critical point of this second stage (Fig. 1). For animal samples, methods and kits utilized are often those already implemented and validated for human DNA, at most protocols have to be adapted according to the type of biological material to be handled. Indications about the starting material treatment are included directly in the manufacturer’s manuals. For botanical evidence, the extraction procedure is more insidious, time-consuming and less standardized than that for animal material. The lack of DNA standardised isolation protocols for plants lies in the high biodiversity of plants and in the extraction efficiencies’ broad variability of different tissues and plant components. However, some casework specific hints on protocols and methods for botanical DNA extraction can be found in the literature [45–47]. A summary of the most popular DNA isolation methods and commercial kits from different types of plant materials are reported in the work of Robertson et al. [47].

As widely established, regions from the mitochondrial genome are the primary choice for forensic taxonomic identification of animals [13, 45–52] due to the high number of mtDNA copies in each cell and the lack of recombination. Moreover, this also due to the fact that the mitochondrial DNA has conserved regions among the different taxa where the PCR primers are located which alternate with other regions with high nucleotide diversity that allow the definition of the species [2, 52]. As illustrated in the flowchart (Fig. 1), depending on the level of identification required by the specific forensic casework, the choice of the locus and of the primer sets to use may differ and this could lead to a deeper level of identification when required.

For animal samples, when the question being asked refers to the identification at species level, the successful amplification of a single marker could be enough to ensure a correct assignment. Therefore, currently, the most used markers for animal species identification are cytb, COI, 16 S rRNA, 12 S rRNA [2]. Furthermore, a 648 bp fragment of COI has been proposed in 2003 with the pioneer work of Hebert et al. for the resolution of the animal kingdom by the DNA barcoding project for taxonomy [47, 53]. The availability of universal primers for a small number of validated markers and the huge production of sequences of many living organisms has ensured that the barcoding strategy had a great consensus internationally. DNA barcoding has been broadly accepted as a reliable method of species identification and the official fragment was introduced also in the forensic field thanks to the high standardisation of the method. However, even though the forensic analysis benefits from DNA barcoding, there are some characteristics of the forensic work that make it necessary to adapt and validate the method to this specific context. In casework situations, the presence of DNA degradation and inhibitors may complicate the recovery of a full-length 648 bp COI barcode sequence for animals. Although generally longer sequences could give greater resolution, also shorter sequences could provide attribution at species level. For this purpose, suitable primers for smaller fragments have been proposed to be used in case of particularly compromised material [12, 54], also within the barcode COI [13]. In some particular cases, when specific species are to be excluded or identified especially in wildlife, the use of taxon specific PCR markers or specific primer sets are required to deepen the level of diagnosis [2, 53–59]. Indeed, the aforesaid situation may arise when it is crucial to identify some biological material at the subspecies level.

Markers used for barcoding animals turned out to be not practical for plants, because the plant genome has evolved quite differently. Ribulose 1,5-biphosphate carboxylase (rbcL) and maturase K (matK) has been proposed as barcode regions to resolve land plants, adopting a multi-locus approach [60]. However, given the complexity of the plant kingdom, supplementary regions from both the chloroplast and the nuclear genome are often required to ensure accurate specimen identification in some groups [15]. In previous works, authors underlined the pitfalls encountered with the application of the official barcode matK to forensic caseworks and then the need of an alternative regions as “forensic” barcodes [14–17]. MatK has high levels of discrimination, but it suffers of limited sequencing success in some groups, as reported in several studies, and often requires the use of specific primers and datasets.

Furthermore, the fragment amplified by the primers indicated by the CBOL is too large to work well in the presence of DNA degradation. Therefore, authors recommended the two plastid markers rbcL + trnH-psbA as the best combination in forensics [16, 17]. However, few subsequent studies have been done to test and confirm the effectiveness of this pair of markers in forensic molecular botany [61] and others tried to test the most suitable markers for forensic plant species identification [62, 63].

When analysing plants in forensics, the possible pathways for the molecular outcome are varied and more complex than for animals, as illustrated in the orange area of the flowchart (Fig. 1). In general, for botanical samples, the selection of the two forensic barcode markers should be the first choice if an identification at species or higher taxonomic levels is enough for the case. When the resolution reached is not adequate to provide the required answers, it is possible to try to deepen the analysis by resorting to species-specific primers for the markers already chosen.

Non-human species identification is only possible if the unknown sample corresponds to a previously well characterized, duly recorded species whose DNA sequence has been submitted to the database.

It is therefore important to have a common standardised marker or few markers whose sequence is adequately represented in databases for the greatest number of species.

The best approach to adopt is to use a validated marker following the indications of published best practices and guidelines. Then, if the database search provides an inconclusive or an ambiguous result, the molecular analysis should be re-attempted with a second marker always among those standardized by the scientific community. Where identification of a certain species or an ambiguous result has to be confirmed or denied, taxon specific markers and primers could be used, either individually or in combination with those in conserved regions [8].

## Data processing and interpretation *(flowchart green area)*

As a final step in molecular species identification, the sequence obtained from a given evidence must be queried against a reference sequence database for the search of the most similar record through alignment. Reference sequences from a private and/or open access public database are used for comparison. A sample will be assigned to a certain taxonomic level, while excluding all other species, genera and families, based on the degree of similarity between the questioned sequence and the reference one. Currently, the two main freely available repositories of sequences are GenBank and BOLD [64, 65]. Hundreds of millions of DNA sequences are publicly accessible, providing reference data of hundreds of thousands of organisms. More recently, whole genome sequences have been uploaded to GenBank, providing a rich array of material for sequence comparisons. BOLD was built by CBOL and collects records only from the official barcode markers for animals, plants and fungi, together with morphological and distributional data. The probability of identification depends on several factors and it is necessary to distinguish between the search for similarity in the animal and in the botanical context. Firstly, like in an instant picture, the rate of success in species assignment depends on the current state of the reference database which is constantly changing as records are updated and new sequences are imported. Moreover, it depends also on the search parameters set by the user [66].

For animals, the process of database searching and the achievement of identification success is simpler. If the corresponding species is registered in the database, the identification is usually quite immediate occurring with the sequence which has brought the first match in the search process [8]. However, close attention to the output provided by the GenBank database is required: the order in which the results of similarity are returned is strictly dependent on the coverage between sequence queries and sequences in the repository. It was experienced that sequences that have lost many bases in the alignment, could present a greater percentage of similarity due to a high coverage and for that reason occupy the first positions of the report, giving a wrong result. In contrast, sequences with less coverage and a complete alignment, among which the correct match could be present, could occupy lower positions in the report due to a lower percentage of identity. Therefore, to have complete databases is fundamental.

Moreover, it was also experienced that plant molecular analysis presents several challenges. For plants, due to the complexity of the plant kingdom, the database coverage in terms of deposited sequences of the currently known species is still incomplete.

The lack of the known corresponding species can lead to an ambiguous or an incorrect (false-positive) species identification, as the comparison is only achievable at higher taxonomic ranks (genus, family, order). A more detailed description of the possible scenarios that may be encountered in plant species identification searching can be found in Ferri et al. [17].

Accurate DNA-based classification of animal and plant material is also critically dependent on the quality of available reference sequences for the taxonomic group of interest. Ideally, all reference sequences contained in either database should have been derived from a vouchered specimen, initially identified by a taxonomic expert. The sequences up-loaded to GenBank are often unregulated and can lead to uncertainty. This point is particularly mandatory in the forensic context, where the quality standards to be respected are particularly strict.

Laboratories that routinely perform DNA-based species identification, especially for wildlife analysis, often have developed an in-house reference database for this purpose, including the sequences of species belonging to the local flora and fauna. The key benefit of an in-house database over a public repository is the ability to directly control and document the suitability of those sequences as reference material for forensic casework, however the number of sequences recorded are usually limited and may not always be representative of the entire variability.

A higher level of curation and control over imported sequences is performed on those deposited in BOLD. Importantly, BOLD does source some data also from GenBank, thus misidentified and erroneous data may however also appear in search results. Moreover, BOLD is restricted only to the official selected barcodes, thus narrowing down the list of markers and sequences that can be consulted. Therefore, it would be important to reach a good point between the need to have the higher number of sequences to increase the probability of positive identification as well as to take care of the quality of the imported records.

Another point to address in order to gain taxonomic identification at species level is the informativeness of the chosen locus, in terms of discriminatory properties. For animals this is a secondary aspect as each marker among those commonly used allows to reach identification at species level with high probability almost always, due to a better definition of species boundaries. For plants, CBOL remarked that even with a multilocus approach, the discriminatory power is to be expected lower than for animals, with a value of 70–80% to the species level [60].

Generally speaking, in plants, the synergy of a less variable, but more widely applicable marker such as rbcL, which can guarantee an identification at higher taxonomic levels, with one more variable such as the intergenic locus trnH-psbA, can help to increase the discriminatory power. However, given the aforementioned great variety of the plant kingdom, it is to be expected that for some specific taxon groups, particularly difficult to distinguish, the use of alternative specific markers may be required. An example of this concerns the genus *Taxus*, as reported in the work of Liu et al. in the peculiar forensic context of bio surveillance [67].

## Discussion and conclusion

Despite the growing interest in this field in recent years, several challenges need to be addressed before NHBT species identification becomes more widely implemented and integrated in the routine investigative process. Whether it’s animal or botanical traces, the genetic identification of species likewise plays a key role in forensic investigation. The main issue is the lack of knowledge and awareness by law enforcement and jury of the high probative potentiality of non-human evidences and DNA-based identification, which is more marked for criminal investigation, rather than for wildlife inspections and especially for botanical evidence analysis.

The consciousness of the botanical evidence is still to be considered unconsolidated. For wildlife traffic investigation, is indispensable the non-human evidence collection and sampling, thus examiners’ awareness about the crucial nature of this first phase in this field has become extremely rooted throughout the years. The illegal wildlife trade, actually, could be considered the most prosecuted crime in many geographical areas [5].

The present work aimed to shed light on the importance of taking into account non-human evidence in forensic science examinations, considering the possibilities and the critical points in order to increase the use of NHFG as an investigative tool. To emphasize the importance of non-human traces in forensic investigations, the European Network of Forensic Science Institutes (ENFSI) has established the Animal, Plant and Soil Traces working group (APST) specialized in casework related analyses [9].

In the course of a crime scene investigation, a broad range of situations may occur, and, in some cases, it may be difficult to pay attention to the collection of non-human findings. Furthermore, botanical traces are often not collected when being fragmented or in minimum amount, erroneously being convinced that recognition is no longer possible or simply underrating their value. To overcome this critical point, more efforts are required to invest time and money in the active training of crime-scene technicians to recognize, collect and properly preserve NHBT. Technically speaking, an important task is to encourage the use of universal and standardized DNA regions and primers approved by the scientific community for species identification and to restrict the use taxonomic specific genomic regions’ fragments and primers only to cases that necessitate more in-depth analysis. This approach would on one hand provide identification based on an alignment with a larger number of sequences, on the other hand it would help to increase the coverage of the database sequences, at least with regard to universal markers.

An additional point to address, actually, is the species coverage of the public databases. About this, the expansion of major public sequence databases is an urgent priority of the scientific community, to include more reference species that might be of interest to forensics specialists. For most wildlife organisms populations genetic data are currently extremely poor in public repositories, referring only to specific genomic fragments. Large and complete databases are of fundamental importance, in particular for the recognition of plants, as the BLAST search algorithm prefers the sequence length in creating the rank of the similarity score, leading to the assignment of the sample to an incorrect species. Moreover, all novel sequences added to the database should be derived from vouchered specimens. Some countries, such as the United Kingdom, have successfully created a ‘library’ of DNA barcodes for a large proportion of their wild plant species [61].

It is also necessary to increase the number of studies that characterize, describe and sequence the population of both flora and fauna, typical of indoor and outdoor environments that most likely can be involved in crime activity. In this way reference local and public databases could be implemented in order to locate unknown NHBT tracks in the context of CSI and standardize the whole process. The lack of a large number of reference species in term of complete, official, controlled and standardized sequences in the public repository GenBank, affects the identification of unknown query species due the poor coverage of marker sequences.

The identification of animal species is facilitated by the smaller ‘population’ of individuals in terms of number present on earth and by their less diversification from the genetic point of view respect to the plant kingdom. Researchers estimate more than 400,000 species of plants living on our planet today. Thus, the problem of poor species coverage proves particularly difficult to deal with in the plant kingdom, not to mention the fact that many species are still unknown. Moreover, even with a total coverage of existing species on earth, some plant species may not be distinguishable at the taxonomic level of species, but only at higher levels (genus, family), as they do not possess sufficient interspecific variability. A successful species identification, hence, depending on the interpretation of database search results, is more challenging for plants than for animals and it deserves particular attention.

The mission of the International Barcode of Life project (iBOL) to build DNA barcode libraries with a large number of reference sequences in order to inventory and assess biodiversity, could also be of help and benefit for forensic applications. Several efforts are needed to draw up protocols with increased sensitivity in order to facilitate the recovery of full target sequences even from low quantity and quality DNA samples and hard matrices (e.g., ivory, teeth, bone, timber, spores, degraded plant fragments).

One of the tasks that still remains challenging at the moment is to overcome the difficulties related to the species identification of degraded and mixed non-human biological material, this is more marked if considering simple, cost-effective, rapid and reliable methods for the analysis. NGS technology for DNA metabarcoding could represent the solution to get over these difficulties, however it has inherent limitations such costs and times that do not allow the simple adoption in routine analysis [68–71]. Some new fluorescent multiplex PCR assay have been implemented to improve standardization of mixed samples identification, although it remains a method applicable to a limited number of detectable species [17]. The standardization of the analytical workflow for plant DNA typing is another crucial matter to work on. Whether for the animal species identification it is possible to take advantage of international recommendations [2], the molecular identification applied to forensic botany is much less defined in terms of accepted protocols and indications as a guide for the analyst dealing with this type of material. Molecular analysis of plant species, more than the animal counterpart, is affected both by abeyance to be considered as an exhibit or clue, and by a limited use related to a greater difficulty of treatment and analysis.

The difficulty to use the already validated protocols for animals also in botanical evidence evaluation lies in the complex nature of the plant kingdom itself, requiring dedicated strategies.

There may therefore be a misconception that the molecular analysis aimed at the forensic identification of botanical species in criminal context has a relatively simple analytical workflow, as the one widely validated by scientific society for the distinction of human material from animal material of domestic or wild origin.

Authors underline the need to share and publish more real casework examples of the utility of the use of non-human evidence in forensic investigations, especially for the plant counterpart. Institutions should make concrete efforts to encourage initiatives that promote quality and innovation in the NHFG field. The combination of ‘traditional’ approaches such anatomy and morphology observation, especially for plants, with DNA-based technology has the potential to enhance the implementation of non-human species identification in forensic investigative processes.

Finally, active collaborations and partnerships between forensic scientists are also necessary to bridge the current gaps, align methodologies, allow the exchange of knowledge and expertise and work on the implementation of standardized procedures.

## Data Availability

Not applicable.
